# The occurrence of extended-spectrum β-lactamase producing *Shigella* spp. in Tehran, Iran

**Published:** 2013-06

**Authors:** Reza Ranjbar, Farzaneh Mirsaeed Ghazi, Shohreh Farshad, Giovanni Maurizio Giammanco, Aurora Aleo, Parviz Owlia, Nematollah Jonaidi, Nourkhoda Sadeghifard, Caterina Mammina

**Affiliations:** 1 Molecular Biology Research Center, Baqiyatallah University of Medical Sciences, Tehran, Iran; 2 Department of Biochemistry, Payam Noor University, Tehran, Iran; 3 Professor Alborzi Clinical Microbiology Research Center, Shiraz University of Medical Sciences, Shiraz, Iran; 4 Department of Sciences for Health Promotion ‘‘G. D'Alessandro’’, University, Palermo, Italy; 5 Department of Microbiology, Faculty of Medicine, Shahed University, Tehran, Iran; 6 Health Research Center, Baqiyatallah University of Medical Sciences, Tehran, Iran; 7 Clinical Microbiology Research Center, Ilam University of Medical Sciences, Ilam, Iran

**Keywords:** ESBLs, *Shigella* spp, Antibiotic resistance

## Abstract

**Background and Objectives:**

The emergence of extended-spectrum β-lactamase (ESBL)-producing *Shigella* spp. is of increasing clinical concern specially in children worldwide. The aim of this study was to investigate the occurrence of extended-spectrum β-lactamase producing *Shigella* spp. in Tehran, Iran

**Materials and Methods:**

The study included all *Shigella* isolates recovered from pediatric patients aged less than 12 years admitted to a major pediatric hospital in Tehran, Iran, from 2008 to 2010. Bacterial identification, antimicrobial susceptibility testing, extended spectrum β-lactamases (ESBLs) screening and confirmatory tests were performed according to the standard guidelines. Conjugal transfer experiments and plasmid analysis were also carried out. Polymerase chain reaction and sequencing were used to identify the genetic determinants responsible for ESBL production.

**Results:**

Four out of 55 *Shigella* isolates, including three *S. sonnei* and one *S. flexneri*, showed an ESBL-positive phenotype. Plasmid transfer of the ESBL phenotype was successful for the *S. flexneri* isolate only. By PCR and sequencing, one *S. sonnei* isolate tested positive for the CMY-59 gene, while the other two *S. sonnei* and the *S. flexneri* isolates tested positive for the *bla*_TEM-1_ and *bla*_CTX-M-15_ genes.

**Conclusion:**

We found the prevalence of ESBL producing *Shigella* isolates was higher than detection rates observed in many other countries. Our finding raise concerns about the dissemination of ESBL among the strains of endemic *S. sonnei* throughout the country, because this species is now the most frequently isolated *Shigella* species in Iran and shigellosis by such strains in the community can pose a significant threat to patients and presents a challenge for disease management.

## INTRODUCTION

Infections caused by *Shigella* species remain a major cause of diarrheal disease associated with high morbidity and mortality in developing countries, such as Iran (1). Among the four *Shigella* spp., *S. sonnei*, is the most commonly isolated species particularly in industrialized countries (2).

Adequate antibiotic therapy is helpful in shigellosis because it may shorten the clinical course of illness, significantly reduce the risk of transmission and prevent also potentially lethal complications. However, high frequencies of resistance to commonly used antimicrobial agents have been reported worldwide in *Shigella* spp. Moreover, the emergence of resistance to third-generation cephalosporins (3GC) in *Shigella* spp. is a matter of great public health concern, mainly in developing countries (3).

Since the initial report of SHV-11–type β-lactamase in *S. dysenteriae* from India in 1999, *Shigella* species producing CTX-M, SHV, and TEM-type extended spectrum β-lactamases (ESBLs) have been identified worldwide from different regions (4). Indeed, an increasing number of countries, including United States, France, Turkey, Lebanon, Bangladesh, Hong Kong and China have recently notified the isolation of ESBL-producing *Shigella* isolates (5–11).

There are several reports showing that the frequency of resistance against different antibiotic groups other than third-generation cephalosporins is increasing among the *Shigella* strains isolated in Iran (12–15).

Here we report the identification and characterization of ESBL producing *Shigella* strains in Tehran, Iran.

## MATERIALS AND METHODS

### Bacterial isolates and antimicrobial susceptibility

The study included all *Shigella* isolates recovered from pediatric patients aged less than 12 years and admitted to Children's Medical Center in Tehran, Iran, from November 2008 to May 2010. A single specimen was obtained from each patient, and rectal swabs were collected from patients on the day of admission at the hospital. All strains were identified at a genus level by conventional methods according to previously described procedures (16). Serotyping of the isolates was performed by slide agglutination (*Shigella* Antisera, Denka Seiken, Tokyo, Japan).

Antibiotic susceptibility testing was performed according to CLSI guidelines (17) using the following antibiotic disks (Oxoid, Basingstoke, Hampshire, UK): ampicillin (AM, 10 µg), amoxicillin–clavulanic acid (AMC, 20–10 µg), cephalotin (CF, 30 µg), cefixime (CFM, 5 µg), cefotaxime (CTX, 30 µg), ceftizoxime (CT, 30 µg), ceftriaxone (CRO, 30 µg), ceftazidime (CAZ, 30 µg), ciprofioxacin (CIP, 5 µg), chloramphenicol (C, 30 µg), doxycycline (D, 30 µg), gentamicin (GN, 10 mg), kanamycin (K, 10 µg), nalidixic acid (NA, 30 µg), neomycin (N, 30 µg), nitrofurantoin (FD, 300 µg), piperacillin (PIP, 100 µg), streptomycin (S, 10 µg), tetracycline (TE, 30 µg), ticarcillin (TIC, 75 µg), tobramycin (TOB, 10 µg), trimethoprim–sulfamethoxazole (SXT, 1.25-23.75 µg).

### Detection and characterization of ESBLs

Screening of 3^rd^ generation cephalosporins (3GC) resistant *Shigella* isolates was performed by disk diffusion method using ceftazidime, cefotaxime, ceftriaxone and cefepime disks. According to CLSI criteria for detection of ESBL producers, each isolate with inhibition zone diameter ≤22 mm for ceftazidime or ≤27 mm for cefotaxime was considered as a potential ESBL-producer. Phenotypic confirmation of ESBL production was done by double disk synergy and combination disk tests (18).

*Escherichia coli* ATCC 25922 and previously characterized ESBL-positive *E. coli*, *Klebsiella pneumoniae* and AmpC-positive *Enterobacter cloacae* strains were used as controls for antibiotic susceptibility testing and the following ESBL analysis. Phenotypic detection of AmpC enzymes was performed by using cefoxitin supplemented with cloxacillin and *E. coli* ATCC 25922 according to the previously described method (19). In brief, the 30 µg cefoxitin disk was supplemented with 200 µg of cloxacillin (Sigma-Aldrich, Singapore). A strain was considered to be an AmpC producer when it showed an increase in diameter around the antibiotic disk with added cloxacillin compared to that with the cefoxitin disk alone.

A multiplex PCR assay approach was used for a rapid screening of frequently encountered β-lactamases according procedure previously described by Dallenne *et al*. (20). DNA of the isolates under study was subjected to the multiplex PCR reactions I to III in 50 µL reaction mixtures containing variable concentrations of specific-group primers and 1 U of *Taq* polymerase (20). Amplification was carried out as follows: initial denaturation at 94°C for 10 min; 30 cycles of 94°C for 40 s, 60°C for 40 s and 72°C for 1 min; and a final extension step at 72°C for 7 min. Amplicons were visualized on 2% agarose gels.

To identify the β-lactamase genes detected in the multiplex PCR assays, amplicons were purified using a QIA quick PCR Purification Kit (Qiagen, GmbH, Germany) and bidirectional sequencing was performed. The nucleotide sequences and the deduced protein sequences were analyzed with the BLAST and Clustal W programs (multiple sequence alignment, pairwise comparisons of sequences and dendrograms).

Resistance transfer experiments were performed for all four isolates as previously described with the rifampicin resistant *Escherichia coli* K12J5 as the recipient. Briefly, mixed cultures of the donors and the rifampicin resistant recipient *E. coli* strain were incubated at 37°C overnight. The transconjugants were selected on MacConkey agar plates supplemented with ceftazidime or cefotaxime- 30 µg/ml - and rifampicin - 250 µg/ml.

A confirmatory test for ESBL and AmpC phenotypes and PCR detection for ESBL genes were performed on transconjugant colonies by the above mentioned procedures. Plasmids from *Shigella* isolates and *E. coli* transconjugants were extracted by an alkaline lysis extraction method (21).

## RESULTS

Fifty five *Shigella* isolates were isolated during the study period. Of theses, 51 strains (92.7%) were susceptible to 3GC. As shown in Table 1, four strains (7.3%), including three *S. sonnei* and one *S. flexneri* showed concomitant resistance to these 3GC antibiotics and were selected for ESBL production.

**Table 1:** Characteristics of the ESBL-producing *Shigella* strains under study.

Strain No.	Time of isolation	Serotype	Antibiotic resistance pattern*	Type of β-lactamase
1	June 12, 2009	*S. sonnei*	AMC-AM-NA-CAZ-TIC-CTX-CRO-CT-PIP-D-TE-S-SXT-CF	CMY-59
2	July 23, 2009	*S. flexneri*	AMC-AM-NA-CAZ-TIC-CTX-CRO-D-TE-S-SXT-C-CF	TEM-1, CTX-M-15
3	September 3, 2009	*S. sonnei*	AMC-AM-NA-CAZ-TOB-TIC-CTX-CRO-PIP-D-TE-S-SXT	TEM-1, CTX-M-15
4	November 10, 2009	*S. sonnei*	AMC-AM-CZA-TOB-TIC-CTX-CRO-D-TE-S-SXT-CF	TEM-1, CTX-M-15

* AM: ampicillin, AMC: amoxycillin-clavulanic acid, C: chloramphenicol, CAZ: ceftazidime, CF: cephalothin, CRO: ceftriaxone, CT: ceftizoxime, CTX: cefotaxime, D: doxycycline, NA: nalidixic acid, PIP: piperacillin, S: streptomycin, SXT: trimethoprim-sulfamethoxazole, TE: tetracycline, TIC: ticarcillin, TOB: tobramycin

Double disk synergy and combination disk tests confirmed that these four strains had ESBL-positive phenotype (Table 1). *bla*_TEM_ and *bla*_CTX-M_ sequences were detected in the *S. flexneri* and two *S. sonnei* isolates, whereas the third *S. sonnei* isolate showed a *bla*_CMY_ sequence. DNA sequencing revealed that the *bla*_TEM_ and *bla*_CTX-M_ sequenceswere *bla*_TEM-1_ and *bla*_CTX-M-15_, respectively, whilst the CMY ESBL was identified as CMY-59.

**Fig. 1:**
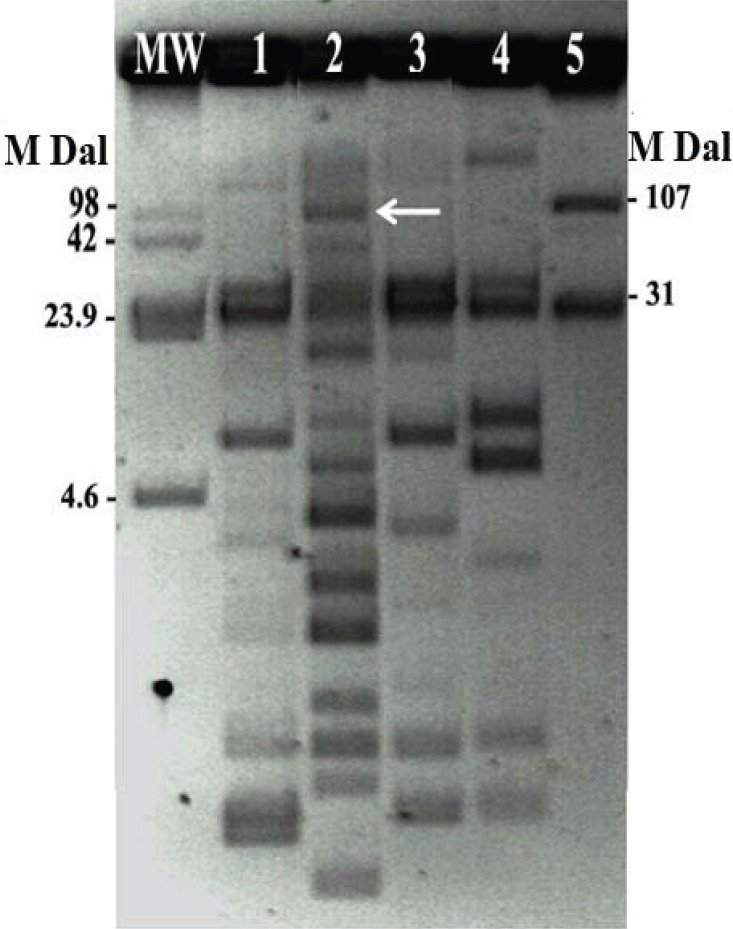
Plasmid patterns of the *Shigella* clinical isolates and the transconjugant *Escherichia coli* from the *S. flexneri* isolate. Lanes: MW, molecular weight, *Escherichia coli* 39R861; 1, *S. sonnei* n.1; 2, *S. flexneri* n.2; 3, *S. sonnei* n. 3; 4, *S. sonnei* n.4; 5, *E. coli* K12J5 transconjugant from the *S. flexneri* isolate n.2.

Based on the conjugation assay, only the *S. flexneri* isolate was able to transfer the *bla* gene to transconjugant, confirming that it was mediated by a plasmid of approximately 107MDa (Fig. 1). Only resistance to trimethoprim was co-transferred along with the ESBL encoding sequence.

## DISCUSSION

Ampicillin and sulfamethoxazole-trimethoprim are generally indicated as the first-line drugs for therapy of shigellosis. However, in the event of disease caused by a multidrug resistant strain, second-line drugs including 3GC and fluoroquinolones are used for treatment of children and adults, respectively (7). Unfortunately, it has been recently reported that the prevalence of resistance to some commonly used antibiotics is increasing in *Shigella* species in Iran (22). Class 2 integrons have proved to be at least in part responsible for the antibiotic resistance spectrum (12).

Beta-lactams are the most frequently used antimicrobial agents in Iran and the ESBL producer's organisms have been reported frequently in several bacterial species in this country (23). Recently Tajbakhsh and her colleagues have documented the presence of *bla*_CTX-M-15_ and *bla*_CMY-2_ in six *Shigella* strains isolated from patients admitted to Milad Hospital, Tehran, Iran (24).

An increase in the occurrence of antimicrobial resistance, including resistance to 3GC and fluoroquinolones among *Shigella* spp. has been observed in several countries. To date, *bla*_CTX-M-15_, *bla*_CTX-M-14_, *bla*_CTX-M-3_ and *bla*_CMY-2_ genes have been reported in *Shigella* spp. isolates (7, 25, 26).

In our study, the prevalence of ESBL producing *Shigella* isolates, accounting for 7.3% of all *Shigella* isolates identified in the period under study, is higher than detection rates observed in many other countries (5, 7, 9). Moreover, CMY-59 has never been reported in *Shigella*, whereas CMY-2-type AmpC ß-lactamases has been previously detected, but infrequently. Recently, the detection of AmpC β-lactamase producer strains in *Shigella* spp. has been reported in Iran (24).

Among all ESBL producing strains, only the *S. flexneri* isolate was able to transfer the *bla* gene to transconjugant, indicating it was mediated by a plasmid however we did not make further attempts to obtain the transfer of resistance determinants. This could have resulted from the presence of chromosomally-mediated β-lactamase genes in the parental strains that could not be transferred to the recipients. Alternatively, they can be located on large low-copy number plasmids with a low transfer frequency.

A ceftriaxone resistant *Shigella* strain with a plasmid mediated CMY-2 AmpC type ESBL was also responsible for a dysentery outbreak in Taiwan (26). On the contrary, CTX-M-15 are the most common types of cefotaximases identified among *Shigella* isolates (3, 5, 6, 27, 28). Of special concern, plasmid mediated production of this β-lactamase is widely disseminated in *Enterobacteriaceae*, particularly among strains isolated from nosocomial infections, but more and more frequently from community-acquired cases.

Our findings raise concerns about the dissemination of ESBL among the strains of endemic *S. sonnei* throughout the country, because *S. sonnei* is now the most frequently isolated *Shigella* species in Iran and shigellosis by such strains in the community can pose a significant threat to patients and presents a challenge for disease management. Thus, active and continued surveillance of antibacterial drug resistance appears to be needed as the first step, along with a judicious use of antibiotics, to minimize the spread of ESBL producing *Shigella* isolates. This is especially critical for the pediatric patients for whom antibiotic therapeutic options are inherently limited.
